# Separating Probability and Reversal Learning in a Novel Probabilistic Reversal Learning Task for Mice

**DOI:** 10.3389/fnbeh.2019.00270

**Published:** 2020-01-09

**Authors:** Jeremy A. Metha, Maddison L. Brian, Sara Oberrauch, Samuel A. Barnes, Travis J. Featherby, Peter Bossaerts, Carsten Murawski, Daniel Hoyer, Laura H. Jacobson

**Affiliations:** ^1^Sleep and Cognition, The Florey Institute of Neuroscience and Mental Health, Parkville, VIC, Australia; ^2^Translational Neuroscience, Department of Pharmacology and Therapeutics, School of Biomedical Sciences, Faculty of Medicine, Dentistry and Health Sciences, The University of Melbourne, Parkville, VIC, Australia; ^3^Brain, Mind and Markets Laboratory, Department of Finance, Faculty of Business and Economics, The University of Melbourne, Parkville, VIC, Australia; ^4^Department of Psychiatry, School of Medicine, University of California, San Diego, La Jolla, CA, United States; ^5^Behavioral Core, The Florey Institute of Neuroscience and Mental Health, Parkville, VIC, Australia; ^6^Department of Molecular Medicine, The Scripps Research Institute, La Jolla, CA, United States

**Keywords:** reinforcement, probabilistic, discrimination, reversal, learning, mouse, cognition, operant

## Abstract

The exploration/exploitation tradeoff – pursuing a known reward vs. sampling from lesser known options in the hope of finding a better payoff – is a fundamental aspect of learning and decision making. In humans, this has been studied using multi-armed bandit tasks. The same processes have also been studied using simplified probabilistic reversal learning (PRL) tasks with binary choices. Our investigations suggest that protocols previously used to explore PRL in mice may prove beyond their cognitive capacities, with animals performing at a no-better-than-chance level. We sought a novel probabilistic learning task to improve behavioral responding in mice, whilst allowing the investigation of the exploration/exploitation tradeoff in decision making. To achieve this, we developed a two-lever operant chamber task with levers corresponding to different probabilities (high/low) of receiving a saccharin reward, reversing the reward contingencies associated with levers once animals reached a threshold of 80% responding at the high rewarding lever. We found that, unlike in existing PRL tasks, mice are able to learn and behave near optimally with 80% high/20% low reward probabilities. Altering the reward contingencies towards equality showed that some mice displayed preference for the high rewarding lever with probabilities as close as 60% high/40% low. Additionally, we show that animal choice behavior can be effectively modelled using reinforcement learning (RL) models incorporating learning rates for positive and negative prediction error, a perseveration parameter, and a noise parameter. This new decision task, coupled with RL analyses, advances access to investigate the neuroscience of the exploration/exploitation tradeoff in decision making.

## Introduction

To survive and thrive in an ever-changing world, both human and non-human animals must make a multitude of rapid decisions about how they interact with, and adapt to, the environment around them in order to optimize gains and minimize losses associated with their behaviors. One large component influencing these decisions is the explore–exploit trade off (Addicott et al., 2017)– to pursue the current best option (exploit), or to test alternative options in the hopes of finding something better (explore). Importantly, exploitative and exploratory behavior must be appropriately balanced to maximize optimal long-term behavior (Addicott et al., 2017).

Probabilistic Reversal Learning (PRL) is a powerful behavioral task which has been used to assess this trade off, as well as cognitive flexibility, impulsivity, and compulsivity. PRL allows the evaluation of how positive or negative feedback differently affect learning in a range of neurological and psychological conditions including Autism Spectrum Disorder, Schizophrenia and Huntington’s Disease in both patient populations (Lawrence et al., 1999) and animal models of disease (Amitai et al., 2014; Amodeo et al., 2014). Additionally, PRL tasks have been used more generally to understand the neurobiological systems and neurotransmitters governing these behaviors (Cohen, 2008; Bari et al., 2010; Costa et al., 2015).

A PRL task involves subjects making a series of choices between binary options, one with high and low probabilities, respectively (e.g., in both rodent and human PRL task, 80% and 20% reward contingencies are commonly used (Mehta et al., 2001; Bari et al., 2010; Dalton et al., 2014)). Subjects are free to choose between the two options and are expected to quickly acquire a preference for the high rewarding option. Once this preference has been established (typically, in the rodent version, as evidenced by eight consecutive choices of the high value option) reward contingencies are reversed; the high value option becomes low value and vice versa. These reversals take place with no additional cues, and in order to successfully engage in the task, subjects are required to do three things. Firstly, subjects must learn to discriminate between the high and low rewarding lever option and learn to favor the high reward lever. Secondly, following a reversal, subjects must realize that the value of the previously high rewarding option has shifted, and lastly, disengage from responding at this previous high value choice and shift toward preferring the other, now high value, option.

Experiments in rats have shown an average peak of three reversals per session in sessions of 200 trials (Bari et al., 2010; Dalton et al., 2014, 2016), and an average peak of six reversals per session in sessions of 600 trials (Amitai et al., 2014), whereas in mice, an average peak between one and two reversals per session in sessions of 400 trials has been reported (Milienne-Petiot et al., 2017).

**FIGURE 1:**
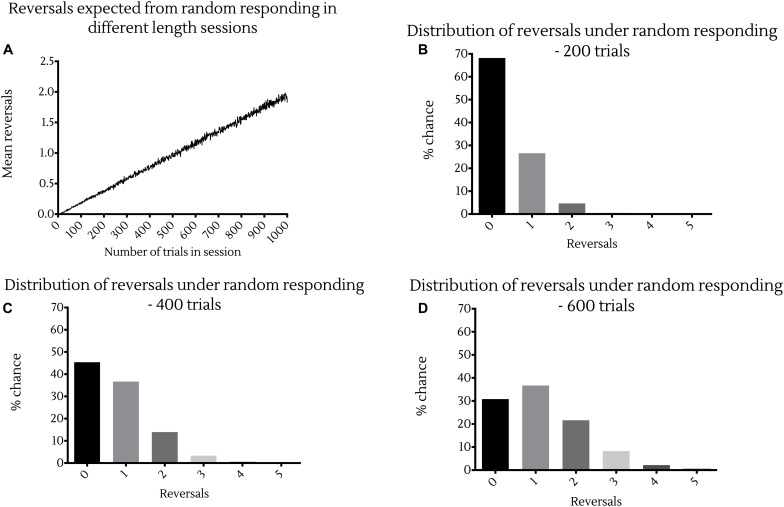
Probability of reversal under random responding with different session lengths. **(A)** Expected reversals averaged across 10,000 simulations of session lengths from 1 to 1,000 trials. Probability distributions showing the likelihood of a certain number of reversal occurring in a session under random responding from 1,000,000 bootstrapped random samples of sessions of **(B)** 200 trials, **(C)** 400 trials or **(D)** 600 trials in length.

To put this in perspective, simulations suggest that the expected number of reversals made by an animal responding randomly in these PRL tasks increases linearly with the number of trials presented in a session (Figure 1A). More precisely, random responding would result in at least three reversals in sessions of 200 trials in 0.55% of cases (Figure 1B), and at least six reversals in sessions of 600 trials in 0.076% of cases (Figure 1D). Conversely, random responding in sessions of 400 trials would result in at least one reversal in 55% of cases, and at least two reversals in 18% of cases (Figure 1C). Together these data indicate that mice perform this task substantially worse than rats, and in a manner much closer to random responding.

To account for this comparatively poor performance, other groups have developed simplified mouse probabilistic learning tasks (Ineichen et al., 2012) in which the high value choice maintains an 80% probability of reward, but choosing the low value option will never provide reward. After sufficient training this presentation of the PRL task is one which mice can easily achieve, with animals making between four and five out of seven possible reversals in a 60 trial session, which is much greater than the 1–2 reversals in 400 trials of mice in the PRL task optimized for rats. However, this simplification changes the task in two major ways. Firstly, it makes it easier to inhibit responding at the previously high rewarding option, as perseverating at this option provides no value at all, compared to the required detection of reduction in value in the full PRL task. And, secondly, it requires only a single rewarded response at the high value option to establish without a doubt that it is indeed the high value option. In both cases, the result is a reduction of uncertainty. Combined, these changes result in a task that is much closer to deterministic reversal learning, in which there is no uncertainty of reward, than to probabilistic learning.

In the present study, we established a task with a different simplification. Rather than altering the probabilistic nature of the task, we focused on allowing mice to acquire a clear preference for the high value option first in an initial block of probabilistic learning sessions, then subsequently reversed the reward contingencies and allowed multiple sessions for animals to inhibit this preference and to learn the new reversed reward contingencies. We found that most animals trained to respond in an operant task were able to successfully acquire an initial preference and subsequently reverse that preference with 80%/20% high/low reward probabilities. As such, we propose that this modified PRL task with 80%/20% reward probabilities would be suitable in subsequent studies to investigate the neurobiology of reinforcement and reversal learning in mice. Additionally, we presented animals with increasingly noisy reward probabilities (70%/30% and 60%/40% on high/low rewarding levers, respectively) and found that some mice were also able to discriminate and reverse in these more complex, uncertain environments, illustrating access to a cognitive gradient within the task.

## Materials and Methods

### Animals

A total of sixteen 10-week old male C57BL/6J Arc mice were used. Mice were kept in a 12-h reversed light dark cycle with free access to water and food-restricted to maintain 90% of their free feeding body weight. Behavioral testing occurred daily between the 3rd and 6th hours of the dark phase. Animal experimentation was conducted in accordance with the Prevention of Cruelty to Animals Act and National Health and Medical Research Code of Practice for the Use of Animals and approved by The Florey Animal Ethics Committee (Application 17-035).

### Apparatus

**FIGURE 2:**
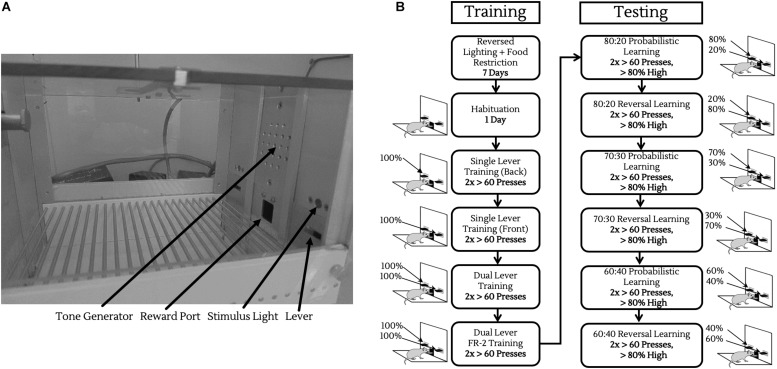
Operant chamber and training/testing protocols. **(A)** Operant chamber fitted with tone generator for reinforcing rewarded trials, liquid reward port for saccharin delivery, two retractable levers, and two stimulus lights located above to indicate when a lever was active. **(B)** Schematic of operant training/testing. Before testing, animals were trained to push levers on both sides of the chamber, and then to push levers twice consecutively for reward (left column). Following this, animals were introduced to probabilistic rewards and reversals, progressively moving to noisier reward contingencies.

All behavioral testing was conducted in 8 operant chambers (Med Associates, St. Albans, VT, United States) enclosed in light and sound attenuating boxes. Each chamber contained two retractable levers, located on either side of a central reward port calibrated to deliver ∼10 μL of sodium-saccharin solution (0.1% w/v, in water) on a rewarding trial, coupled with a 1000 Hz, 75 dB tone. The enclosing boxes were equipped with fans to provide ventilation and to mask extraneous noise, as well as infrared cameras for observing animals during their sessions (Figure 2A).

### Behavioral Tasks

Mice were presented with a single operant session every day throughout the course of the experiment.

#### Habituation

Initially, mice were given a single 30-min session to freely explore the chamber, with no levers presented and ∼50 μL saccharin solution provided in the central reward port.

#### Training

Prior to entering the PRL task, animals underwent several stages of habituation, single lever training on both levers, dual lever training, then dual lever FR-2 training as described below and in Figure 2B.

Throughout training and testing, trials were initiated by the extension of one or both levers in the operant chambers. During each trial, animals had 10 s to make a response at a lever. After responding, or at the end of the 10 s trial if no response was made, the lever was retracted and a 10 s inter-trial interval (ITI) elapsed before the lever extended again and the subsequent trial began.

##### Single lever training

Following habituation, mice were exposed to daily sessions of 100 trials with the lever at the back of the chamber in operation and the lever at the front of the box retracted. If an animal pressed the lever within this window, a reinforcing tone sounded, and a 10 μL saccharin reward was delivered to the central reward port 0.5 s after a successful lever press.

Mice remained in this phase of training with the *back* lever extended until they completed two sessions with greater than 60 rewarded trials and were trained subsequently on the *front* lever until the same criteria were reached.

##### Dual lever training

Next, mice were given sessions with both levers active, with one lever extended at a time, pseudo-randomized to provide five front/back lever extensions per 10-trial block in the 100-trial session. Again, animals remained in this phase of training until two sessions with at least 60 responses were achieved.

##### Dual lever FR-2 training

In previous operant chamber experiments, we observed that mice sometimes made “accidental” lever presses by inadvertent contact with the levers, rather than actively pressing them. To minimize these unintentional responses, we trained animals to press the same lever twice consecutively for reward, using the same randomized lever extensions as in the dual lever training. After achieving two sessions with at least 60 responses, mice progressed into probabilistic learning.

#### Testing

##### Probabilistic learning

In these sessions, both levers were extended simultaneously, with two consecutive presses on the front/back levers providing an 80%/20% chance of reward, respectively. On rewarded trials, levers were retracted, the conditioning tone sounded, and the saccharin reward simultaneously delivered, whereas on unrewarded trials levers were retracted, but with no tone or reward delivery. Mice remained in probabilistic learning until they achieved two sessions with at least 60 responses, and greater than 80% of lever presses made on the high rewarding lever.

##### Reversal learning

Upon achieving criterion in the probabilistic learning task, reward contingencies were reversed; the previously high, 80% reward lever now rewarding on only 20% of trials and vice versa. This task required mice to make a substantial change in their behavior, suppressing their previously learned reward/lever associations and adapting to the new contingencies. As in the probabilistic learning phase, animals remained in reversal learning until they achieved two sessions with at least 60 responses, and greater than 80% of lever presses made on the high-rewarding lever.

##### Further reversal learning

After mice reached criterion in the reversal learning phase, the high and low rewarding levers were again reversed, but now with a 70%/30% probability of reward, rather than 80%/20%. Once mice reached the same levels of performance as above, the levers were again reversed, keeping the 70%/30% reward contingencies. Following completion of reversal learning with the 70%/30% reward contingencies, animals were lastly presented with 60%/40% reward contingencies and reversal.

### Data and Analyses

At each trial, we recorded (a) whether a mouse made a response or not, and whether the chosen lever was associated with high or low reward (high/low/omit), (b) whether a trial was rewarded or not (rewarded/unrewarded), and (c) the time from lever extension to response in milliseconds.

Data were processed in R version 3.6.1 (R Core Team, 2019) with RStudio (Allaire, 2012) using the *tidyverse* package (Wickham, 2016). Choice data were analyzed using reinforcement learning (RL) models (Rutledge et al., 2009; Sutton and Barto, 2018), the parameters of which were estimated on a subject-by-subject basis, and fit by maximizing the likelihood of the observed choices compounded across all trials. These optimizations were carried out using the *optimx* package (Nash and Varadhan, 2011) in RStudio.

#### Analysis of Task Progression

Time taken to reach criterion in different reversal learning stages was compared in a mixed-effects model fitted using restricted maximum likelihood (REML) with days to criterion as main effect and random intercepts for individual animals in order to account for repeated measures.

#### Analysis of Choice Data

We fitted choice and reward data from individual animals to a range of RL algorithms (Sutton and Barto, 2018). These models use the sequence of choices and rewards to estimate and update the expected reward value of each lever on a trial by trial basis (Rutledge et al., 2009; Sutton and Barto, 2018). The expected values were initialized to one as animals were initially trained to push levers for a sure reward at every trial. Model comparisons were made using the relative Akaike Information Criterion (AIC) (Akaike, 1998; Anderson and Burnham, 2002).

Parameters for learning from positive or negative outcomes were compared within animals using a Wilcoxon matched pairs signed-ranks test in Prism 8.0 (GraphPad Software, San Diego, CA, United States).

##### Single learning rate RL model

The simplest model consisted of two parameters; a learning rate α, through which the value of an option is updated following a trial, and the softmax parameter β governing how much the expected values of options affect choices being made. Given expected values *V*_f_(*t*) for the front lever, and *V*_*b*_(*t*) for the back lever, the probability of choosing the front lever *P*_*f*_(*t*) was calculated using the softmax rule:

$$P_{f}\,\left(t\right)=\frac{1}{1+e^{-\beta \,\left(V_{f}\,\left(t\right)-V_{b}\,\left(t\right)\right)}}$$

and following each trial, the expected value of the chosen lever (*V*_*f*_, for example) was updated according to the following rule:

$$V_{f}\,\left(t+1\right)=V_{f}\,\left(t\right)+\text{α}\,\left[R\,\left(t\right)-V_{f}\,\left(t\right)\right]$$

where *R*(*t*)−*V*_*f*_(*t*) is the reward prediction error – the difference between the reward received on a given trial *R*(*t*), and the expected value of the lever *V*_*f*_(*t*).

##### Dual learning rates RL model

This model separated out the α learning rate parameter into two different parameters for positive (α_pos_) and negative (α_neg_) reward prediction errors reflecting the idea that learning rates may be different for positive versus negative experiences (Daw et al., 2002; Frank et al., 2007; Rutledge et al., 2009). In this model, *P*_*f*_(*t*) was calculated in the same manner as in the standard RL model, but following each trial, values were updated according to the rule:

$$V_{f}\,\left(t+1\right)=V_{f}\,\left(t\right)+\left\{ \begin{array}{c} \alpha_{p\,o\,s}\,\left[R\,\left(t\right)-V_{f}\,\left(t\right)\right]|R\,\left(t\right)\geq V_{f}\,\left(t\right)\\ \alpha_{n\,e\,g}\,\left[R\,\left(t\right)-V_{f}\,\left(t\right)\right]|R\,\left(t\right)<V_{f}\,\left(t\right) \end{array}\right.$$

##### Perseverative RL model

This model introduced an additional perseveration parameter δ, which enters into the softmax rule for determining probability of choosing a lever as:

$$P_{f}\,\left(t\right)=\frac{1}{1+e^{-\beta \,\left(V_{f}\,\left(t\right)-V_{b}\,\left(t\right)\right)+\delta \,\left(C_{f}\,\left(t-1\right)-C_{b}\,\left(t-1\right)\right)}}$$

In this model, *C*_*f*_ and *C*_*b*_ are indicator variables, taking the value of 1 if the relevant lever is chosen, and 0 otherwise. A positive δ indicates that an animal is more likely to respond on the same side as the previous trial (perseveration), while a negative δ indicates that animals are more likely to switch from side to side on consecutive trials (alternation).

##### Perseverative dual learning rates RL model

We also fitted a combination of the perseverative and dual learning rates RL models, incorporating α_pos_, α_neg_, β, and δ with *P*_*f*_(*t*) determined by:

$$P_{f}\,\left(t\right)=\frac{1}{1+e^{-\beta \,\left(V_{f}\,\left(t\right)-V_{b}\,\left(t\right)\right)+\delta \,\left(C_{f}\,\left(t-1\right)-C_{b}\,\left(t-1\right)\right)}}$$

and expected reward values updated by:

$$V_{f}\,\left(t+1\right)=V_{f}\,\left(t\right)+\left\{ \begin{array}{c} \alpha_{p\,o\,s}\,\left[R\,\left(t\right)-V_{f}\,\left(t\right)\right]|R\,\left(t\right)\geq V_{f}\,\left(t\right)\\ \alpha_{n\,e\,g}\,\left[R\,\left(t\right)-V_{f}\,\left(t\right)\right]|R\,\left(t\right)<V_{f}\,\left(t\right) \end{array}\right.$$

## Results

### Performance Across Different Stages of Task Progression

**FIGURE 3:**
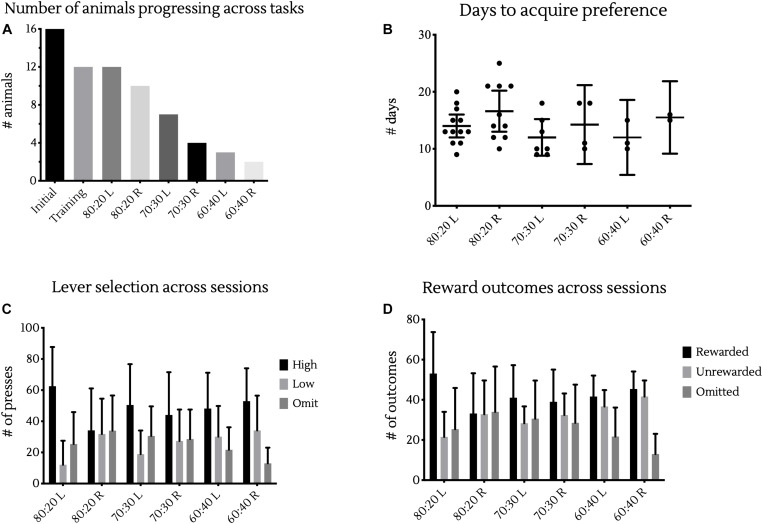
Differences across reward contingencies. **(A)** Bars represent the number of animals able to complete training and each different reward contingency in testing. **(B)** Days to reach criterion in each reward contingency. Points represent the number of sessions individual animals took to achieve >80% of responses at the high rewarding lever in two sessions. Bars represent cohort mean ±95% CI. **(C)** Bars represent the mean ± SD presses in a session made at the high or low levers, as well as the number of response omissions made. **(D)** Bars represent the mean ± SD presses in a resulting in rewards or a lack thereof, as well as the number of omissions made.

From our initial 16 animals, 12 progressed through operant lever training and into the probabilistic learning tasks, all of which were able to reach criterion in the initial 80:20 probabilistic learning phase. Of these 12 animals, 10 were then able to complete the first reversal learning phase. As difficulty increased, fewer animals were able to complete each task, with 7, 4, 3, and 2 animals able to complete the 70:30 Learning, 70:30 Reversal, 60:40 Learning and 60:40 Reversal tasks, respectively (Figure 3A). Despite the increasing complexity of the task, there was no significant difference in the number of days it took animals to reach criterion under different reward contingencies, as analyzed using a mixed-effects model fitted using REML with random intercepts for individual animals (Figure 3B).

### Choice Data Analysis

We fitted data from the 12 animals that progressed into the operant tasks. Of these 12 datasets, the MLE estimation procedure successfully converged to parameter point estimates for the 10 animals able to complete both the 80%/20% Probabilistic Learning and Reversal Learning tasks, with models failing to converge for the two animals unable to complete the 80:20 Reversal Learning task.

In 8 of these 10 mice, the perseverative dual learning rates RL model gave the lowest AIC, and hence the best fit, followed by the perseverative RL model. In the remaining two subjects, this order was reversed, with the perseverative RL model providing the best fit, followed by the perseverative dual learning rates RL model.

**FIGURE 4:**
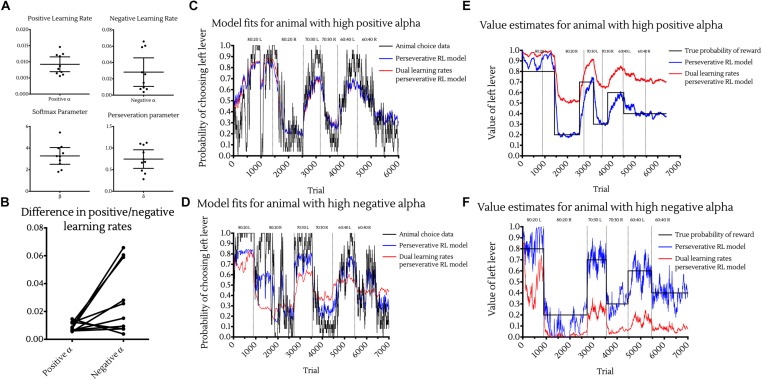
Choice data and reinforcement learning model fits. **(A)** Maximum likelihood estimated parameter values of the Perseverative Dual Learning Rate model. Points represent individual animal estimates. Bars represent cohort mean ±95% CI. **(B)** Individual differences between positive and negative reward prediction error learning rates. Points represent individual animal parameter estimates. Joining lines indicate parameters coming from the same subject. While on average animals had higher α_neg_ than α_pos_ learning rates, this was not apparent in all animals, with much greater variance in negative than positive learning rates. **(C,D)** Observed and model predicted choices for example animals with high positive alpha **(C)** and high negative alpha **(D)** and best fitting models. Choice data were fit with the perseverative reinforcement learning model (blue) and perseverative dual learning rate reinforcement learning model (red) and model predictions plot against the true animal choices using 25-point moving average smoothing (black). Vertical lines indicate changes to reward contingencies. **(E,F)** Value estimates of reinforcement learning models for example animal with high positive alpha **(E)** and high negative alpha **(F)**. Value estimates of one lever from both the perseverative (blue) and dual learning rate perseverative (red) models were plotted against the true probability of reward of that lever (black) over time. Vertical lines indicate changes to reward contingencies.

Investigating the distributions of these parameters across animals resulted in the following population level values (Figure 4A):

• Learning from positive outcomes parameter
α_pos_ = 0.00923 (95% CI : 0.00694, 0.0115).
• Learning from negative outcomes parameter
α_neg_ = 0.0282(95%*CI*: 0.0107, 0.0457).
• Softmax parameter
β = 3.275(95%CI: 2.5, 4.05).
• Perseveration parameter
δ = 0.744(95%CI:0.529, 0.959).

A Wilcoxon matched pairs signed-ranks test showed that α_pos_ was significantly smaller than α_neg_ (*p* < 0.05) and suggests that animals were learning significantly more from negative outcomes than positive ones (Figure 4B). However, this was not observed in all animals, with some exhibiting a higher α_pos_ than α_neg_. Such differences may be expected to occur between individuals and may be suggestive of different underlying neurobiological substrates in learning from positive versus negative outcomes (Daw et al., 2002; Cazé and Van Der Meer, 2013). Additionally, whether an animal showed a higher learning rate to positive or negative outcomes did not impact on its ability to develop preferences for the high value option in the reversal learning tasks. For example, one of the two animals able to perform the most complex 60:40 Reversal Learning task, showed a higher α_pos_ (Figure 4C) whereas the other displayed a higher α_neg_ (Figure 4D).

Although the perseverative dual learning rates model provided the best fit to animal choice data, the *Q*-values generated by this model and used as estimates for the softmax decision rule did not accurately estimate the true probabilities of reward associated with each lever. *Q*-value estimates from models with a greater α_pos_ than α_neg_ were systemically greater than the true reward probabilities (Figure 4E), while *Q*-value estimates from models with a greater α_neg_ than α_pos_ were systemically lower than the true reward probabilities (Figure 4F). Despite this, *Q*-values were accurate on an ordinal level in both situations, with the high rewarding option associated with higher *Q*-values than the low rewarding option.

Interestingly, the perseverative RL model with a single learning rate was better able to accurately track the true probability of reward, with *Q*-values falling much closer to the true probability of reward for each option (Figures 4E,F, blue lines).

## Discussion

We assessed the ability of mice to perform a probabilistic learning task in a two lever operant chamber and determined that: (1) of those animals able to reliably push both levers in the chambers, all were able to “solve” the 80:20 probabilistic learning task, and more than 80% of these animals were also able to reverse under these contingencies; (2) some mice were able to discern and show preference for the high rewarding lever with 70:30 reward contingencies, and a few were even able to solve the highly complex 60:40 learning and reversal tasks; (3) a four parameter RL model, incorporating learning from positive and negative outcomes parameters α_pos_ and α_neg_, softmax parameter β and perseveration parameter δ, was best able to capture animal choice behavior, while a simpler three parameter model with a single α learning rate was able to estimate the true expected values of options across different reward contingencies.

Of those animals capable of reliably pushing both levers in the chambers, all were able to “solve” the 80:20 probabilistic learning task, showing a robust preference for the high rewarding lever after a mean of 14 daily sessions. Additionally, more than 80% of these animals were able to suppress this learned behavior in the 80:20 reversal learning task, showing an equally strong preference for the new high rewarding lever after a mean of 16.6 days. This suggests that mice are able to discriminate reward values and perform PRL when rewards on both the high and low value options are stochastic, although at a much slower rate than rats and/or other species expected to have greater cognitive capabilities. This in turn provides some explanation as to why the performance of mice (Milienne-Petiot et al., 2017) at traditional within-session PRL tasks is so much lower than that of rats (Bari et al., 2010). In within-session PRL tasks, subjects are required to develop and switch preference on the order of 10s of trials, within a single day session consisting of several hundred trials, while our data suggests that mice require >1,000 individual trials to develop these strong preferences and reversals, spread over many separate 100 trial days.

Moving beyond the 80:20 reward contingencies, some mice were able to discern and develop preference for the high rewarding lever in the noisier 70:30 and even 60:40 reward contingency environments (Figures 3C,D). This ability to successfully develop preferences and reverse behavior under the 60:40 reward contingencies is particularly impressive for a number of reasons.

Firstly, it is substantially harder to discern which is the high rewarding side between a 60% and 40% chance of reward than 70%/30% or 80%/20%. Secondly, there is little drive to inhibit the developed preference and explore the alternative option when perseverating at the previously high option will still net almost as many rewards as adapting behavior to the new contingencies. To put this in context, previous progressive ratio studies using saccharin have shown that mice will press a lever upward of 20 times for a single saccharin reward (Beeler et al., 2012), a much lower rate of rewarding than the 40% of the low lever in these situations. It is also interesting to note that there was no significant difference in the time taken to acquire a preference for the high rewarding lever under the different reward contingencies. Were our animals truly behaving according to a RL strategy, we would expect to see acquisition of preference in noisier environments like those in the 60:40 tasks to take longer than in simpler environments like the 80:20 tasks.

One possible explanation of this, is that animals are meta-learning to reverse (that is, increasing task complexity being offset by increasing subject experience/competence), as suggested by Costa et al. (2015) in a monkey reversal learning task. However, given that all our animals received the same reward contingencies and in the same order in this experiment, further work would be required to test these hypotheses.

Lastly, a four-parameter perseverative dual learning rates RL model provided a good fit for choice data from individual animals. Estimated parameters of this model suggest that mice learn more rapidly following negative outcomes than positive outcomes. The positive δ also provides some insight into animal behavior, implying that mice are more likely to perseverate on the same side for multiple trials than to swap from side to side regularly. This δ is quite substantial, accounting for a ∼ 30% increase in the probability of choosing the same option as the trial before, if there was no difference in the expected value of both choices. This drive to perseverate gives further reason as to why mice struggle so much with traditional PRL tasks.

Another point of interest is to compare how these parameter estimates differ from human subjects. A study examining the effects of dopaminergic drugs in Parkinson’s patients and healthy controls (Rutledge et al., 2009) fitted the same perseverative dual learning rates RL model to data from humans performing a dynamic foraging task and found that for healthy young adults the best fitting parameters were α_pos_≈α_neg_≈0.6, β = 1.73 and δ = 0.39. While the human parameters show somewhat smaller β and δ compared to mice, thus implying less noisy behavior and less perseveration, the biggest difference is in the α ’s, with the human parameter orders of magnitude greater than that of a mouse, reflecting a much faster learning rate in humans than mice, as expected.

In addition to providing a good fit to our data, the RL model consisted of only a few, simple, yet highly informative parameters. For example, an intervention which causes an increase in α_pos_ could be interpreted as increasing the salience of reward signals, either by increasing the value of reward, or the rate at which positively reinforced learning occurs, where an intervention causing a decrease in δ might be interpreted as decreasing compulsive, perseverative behaviors. Lastly, this model can serve as an easily adaptable base on which to build other, more complex models with additional parameters; for example an additional updating step could, at the end of a session, help to explain between-session memory consolidation, or a temporal shrinking parameter within-session which might be useful in assessing appetitive satiation/reward devaluation over the course of a session (Isles et al., 2003; Rudebeck et al., 2013).

## Conclusion

This study describes a novel, simplified variant of the PRL task for mice. Rather than removing or simplifying the probabilistic element of the task, we separate the initial discrimination from the reversal learning component. We found that, unlike in the traditional probabilistic learning tasks, mice were able to both acquire an initial preference for a high rewarding probabilistic option, as well as inhibit that acquired preference and subsequently adapt to an altered reward state with very close contingencies. Additionally, we show that RL models provide an appropriate tool for examining choice behavior, offering a framework for evaluating the effects of pharmacological and other interventions on different aspects of probability and reversal learning. However, it is likely that RL is not the complete process governing animal behavior in these tasks as higher order meta-learning or model-based learning processes may be in action. Further experimentation utilizing repeated reversals with the same reward contingencies, or alterations of the order of reward contingencies given, would be required to investigate this aspect further.

## Data Availability Statement

The raw data supporting the conclusions of this article will be made available by the authors, without undue reservation, to any qualified researcher.

## Ethics Statement

The animal study was reviewed and approved by The Florey Animal Ethics Committee.

## Author Contributions

JM, SB, PB, CM, DH, and LJ contributed to the conception and design of the study. JM, MB, SO, and TF collected the data. JM and CM performed the statistical analyses. JM, SB, CM, DH, and LJ wrote the manuscript. All authors contributed to the manuscript revision, read, and approved the submitted version.

## Conflict of Interest

The authors declare that the research was conducted in the absence of any commercial or financial relationships that could be construed as a potential conflict of interest.
